# Relationship between World Assumptions and Post-Traumatic Growth among Polish Cancer Patients: Moderating Effect of Rumination

**DOI:** 10.3390/ijerph191912444

**Published:** 2022-09-29

**Authors:** Małgorzata Szcześniak, Daria Madej, Grażyna Bielecka

**Affiliations:** Institute of Psychology, University of Szczecin, 71-017 Szczecin, Poland

**Keywords:** cancer, oncology, shattered assumptions theory, post-traumatic growth, cognitions, intrusive rumination, deliberate rumination, moderation

## Abstract

Background: Although post-traumatic growth is believed to be the result of complex interplays between various factors, cognitive variables appear to play a special role in these interactions. Yet, research on this topic is scant. Therefore, the first purpose of this study was to verify whether there is a direct relationship between world assumptions and post-traumatic growth among Polish cancer patients. As the effect of psychological change in post-traumatic growth may be affected by basic beliefs about the world and oneself, the second goal was to assess whether this association is moderated by rumination. Methods: The study included 215 Polish cancer patients. The Post-traumatic Growth and Depreciation Inventory—Expanded version—(intrusive and deliberate rumination), the World Assumption Scale, and the Event-related Rumination Inventory were applied. Results: Positive, albeit weak, correlations were found between the dimensions of world assumptions and post-traumatic growth. Post-traumatic growth correlated negatively with intrusive rumination and positively with deliberate rumination. The outcomes show that the level of post-traumatic growth resulting from world assumptions is significantly different at various levels of intrusive and deliberate rumination. Conclusion: Lower/medium intrusive rumination seems to strengthen the relationship between benevolence of the world/worthiness of the self and post-traumatic growth, and higher deliberate rumination tends to reinforce the relationship between meaningfulness and post-traumatic growth.

## 1. Introduction

Cancer is a large and complex group of diseases [1] involving a life-altering or potentially life-threatening experience [2,3]. People diagnosed with cancer and patients undergoing cancer-related treatment commonly report increased psychological distress [3], fatigue [4,5], cognitive deficits [6], and physical problems [7]. Cancer is also a serious public health problem [8], associated with economic and financial burden [9]. According to World Cancer Research Fund International, more than 18 million people were diagnosed with cancer in 2020 [10]. The World Health Organization confirmed that nearly 10 million people died of cancer in 2020 [11], making it the second-leading cause of death globally, after cardiovascular diseases [12]. Cancer incidence rates in Poland are lower than in other EU countries, but cancer mortality is higher [13,14], thus becoming a growing health and social problem [15].

Even though cancer is a stressful, challenging, and traumatic event [3], this difficult experience may paradoxically have some positive consequences among cancer survivors [16,17]. In fact, Henson et al. [18] observed that the growth and positive psychological changes are not an outcome of the event itself, but rather a consequence of several components. In the group of main factors that affect post-traumatic growth, researchers mentioned personality, socio-cultural elements, interpersonal experiences [19], social support [20], religious beliefs [20], emotions, and cognitions [21]. Although post-traumatic growth is considered to result as an effect of complex interactions [20], cognitive factors seem to play a special role. Yet, research on this topic is scant [22]. Therefore, in this study, the first purpose was to verify whether there is a direct relationship between dimensions of world assumptions and post-traumatic growth among Polish cancer patients. The second aim was to analyze the relationship between components of rumination and post-traumatic growth. Clinical practice has confirmed that cognitive schemas after traumatic life events help in their confrontation; thus, they are relevant for broadly understood effective functioning [23]. Likewise, because the effect of psychological change in post-traumatic growth may be affected by basic beliefs about the world and oneself [24], the third goal was to assess whether the level of post-traumatic growth resulting from world assumptions is significantly different at various levels of intrusive and deliberate rumination. In other words, the third aim was to explore whether this association is moderated by rumination. This repetitive and recurrent thinking has been found to be linked to cognitions [25] and post-traumatic growth [26].

### 1.1. Post-Traumatic Growth

The concept of post-traumatic growth refers to “the subjective experience of positive psychological change reported by an individual as a result of the struggle with trauma” [27] (p. 628). According to Tedeschi and Calhoun [28], post-traumatic growth perceived as a “transformative development” [29] (p. 6) often coexists with personal distress. As such, post-traumatic growth is not a mechanical response to a traumatic event, and numerous factors affect positive life changes [30]. However, people differ in their approach to difficult situations. The same event may have negative effects in some individuals, such as withdrawal or depressive symptoms. In turn, other individuals adapt to the situation and see it as an opportunity for growth [31]. Consequently, some people may experience post-traumatic growth after going through traumatic events, while others may not [32]. Post-traumatic growth encompasses stronger interpersonal relationships, new opportunities in life, greater appreciation of one’s existence, and/or more mature religious or spiritual development [33].

### 1.2. World Assumptions

According to the widely recognized theory of shattered assumptions [34,35,36], there are three major categories of beliefs. The first category consists of assumptions about the world’s benevolence. Most people present an optimistic way of explaining events based on the belief that the world around them is a good and safe place, and other people are kind and caring [34,36]. The second category reflects the meaningfulness of the world and the allocation of outcomes [34]. Human understanding is guided by three principles: justice, controllability, and randomness [34,37]. First, people assume that they “deserve what they get and get what they deserve” [34] (p. 118). Second, people engage in appropriate behavior to minimize negative effects and their own vulnerability. Third, people believe that the outcome is a consequence of chance, luck, or fortune [34,37]. The third category implies the worthiness of the self and indicates an individuals’ beliefs about their own integrity.

### 1.3. Post-Traumatic Growth and World Assumptions

A comprehensive model of post-traumatic growth [31] suggests that assumptive world beliefs affect the way people interpret a life-threatening event and cope with stress. More specifically, basic assumptions about the world and the self may influence human thinking, feeling, and acting, giving people a sense of agency [34,35]. Calhoun et al. [38] considered cognitive processing an essential element of an individuals’ efforts to restore their perspective and adjust to a difficult situation. Going through cancer threatens a set of these assumptions and results in cognitive processing and schema modification. Thus, patients may implement or reconstruct new systems of belief to adapt to the given experience [39].

Consistent with the theoretical model, there is some empirical evidence linking post-traumatic growth among cancer patients with a phenomenon conceptually similar to world assumptions. For example, post-traumatic growth correlates with the search for and presence of meaning [40,41], meaning-focused coping [42], resilience [43], domains of intrusion [43], optimism [44,45], openness to experience [46], reappraisal of worldviews [42], benefit finding [42], self-worth [47], and hope [45]. Moreover, in other studies, the assumptions of justice and luck, cognitive avoidance [40], self-controllability [48], core belief challenges [49], and perceived social support [49] predicted post-traumatic growth.

### 1.4. Intrusive and Deliberate Rumination

Rumination is considered a way of coping [50] in which people react to stress [51] by recurrently reflecting on an event [52]. This metacognitive process [53] can be classified as intrusive and deliberate [54]. Intrusive rumination, defined as the unconscious repetitive thinking about a negative and stressful situation [55], manifests itself in experiencing unconstructive emotional states. This depressive type consists of persistent thinking about the causes, meaning, and repercussions of negative past events [56,57]. High ruminators do not solve problems actively [56], remain fixated on the source of the difficulty [56], and tend to be dysfunctionally self-focused [58,59]. Rumination is associated with deficits in cognitive control [60], attentional inflexibility [52], various undesirable personality traits [56], and the worsening of negative moods [61].

Although rumination is often perceived as negative, intrusive, automatic, brooding, and disruptive, the multidimensional perspective of this concept also takes into account its positive aspects [22,62]. For example, Martin and Tesser [63] (p. 9) define rumination as a “class of thinking.” According to this operationalization, people may ponder positive and negative content. Moreover, they can reflect on the past, the present, and the future. In this sense, rumination has a deliberate, purposeful, and constructive character [62]. Additionally, deliberate rumination may help in facing difficult situations, reducing the negative effects of traumatic events, and actively searching for solutions to problems [55].

Experiential and empirical evidence shows that the initially traumatic situation of cancer may lead people to shattered world assumptions with intrusive thoughts. Some patients become inactive due to the trauma and do not take any action to make changes in their lives [22]. With time, many of them are able to comprehend and explain their own experience in a new way [64].

### 1.5. Rumination as a Potential Moderator

Rumination, as stated above, is considered to be a way of coping [50] in which people react to stress [51] by recurrently reflecting on an event [52]. Its depressive type consists of persistent thinking about the causes, meaning, and repercussions of negative past events [56,57].

Past research has shown that three main types of factors are related to post-traumatic growth: the features of the event, personal qualities, and cognitive processing. Rumination seems to play a crucial role in post-traumatic growth [65]. In fact, Su and Chen [49] confirmed that people with deliberate rumination tend to display post-traumatic growth because the re-examination of difficult events (e.g., cancer or other highly challenging situations) helps in their positive reassessment. Deliberate or reflective thoughts have been found to be related to lower levels of depression [53]. Moreover, rumination has been analyzed as a moderator in the relationships between depression assessed at baseline and six months later [66], mistreatment and depersonalization [67], work interruptions and well-being [68], and mindfulness and distress reduction [69]. It is plausible that rumination may alter the direction or the strength of the relationship between core beliefs and post-traumatic growth. Thus, based on the abovementioned evidence, it can be expected that not all patients with world assumptions display the same levels of post-traumatic growth.

Based on the theoretical premises and empirical results of other studies, we assumed that:

**Hypothesis** **H1** **(H1).** *World assumptions positively correlate with post-traumatic growth*.

**Hypothesis** **H2** **(H2).** *Post-traumatic growth correlates negatively with intrusive rumination, and positively with deliberate rumination*.

**Hypothesis** **H3** **(H3).** *The level of post-traumatic growth resulting from world assumptions is significantly different at various levels of intrusive and deliberate rumination*.

In the present study, world assumptions were variable X, post-traumatic growth was variable Y, and both types of ruminations (intrusive and deliberate), W, were single moderators.

## 2. Materials and Methods

### 2.1. Participants and Procedure

The study included 215 Polish cancer patients (Table 1) who voluntarily enrolled in the study and were asked to fill out the self-report instruments. The inclusion criteria were adults aged over 18 years diagnosed with any type of cancer, and willing to participate in the research. After explaining the general aim of the research, they provided informed written consent to take part in the research. The study was carried out in the period from February 2022 to June 2022. The participants filled in the paper–pencil questionnaires at the Oncology Department of the Nicolaus Copernicus Provincial Hospital in Koszalin and at Pomeranian Medical University in Szczecin.

The age range of the sample was 18–91. Most of the participants were women. In the case of marital status, most respondents were unmarried, followed by informal and married couples. The fewest respondents were divorced participants. In terms of education, people with a higher education level prevailed. The following types of cancer were diagnosed among the respondents: lung, breast, pancreatic, esophageal, stomach, prostate, testicular, liver, and gynecologic, such as uterine or ovarian. The mean time from diagnosis (denoted from here on as timing), calculated in months, was *M* = 20.59 (*SD* = 15.06).

The study protocol was accepted by the Bioethics Committee of the Institute of Psychology at the University of Szczecin (KB 01/2022, 12 January 2022), and the research was performed in accordance with the Declaration of Helsinki.

### 2.2. Post-Traumatic Growth and Depreciation Inventory (PTGDI-X)

The Post-traumatic Growth and Depreciation Inventory—Expanded version—in its Polish adaptation by Taku et al. [70] was used to measure the intensity of post-traumatic growth (PTG) and post-traumatic depreciation (PTD) among the cancer patient participants. The PTGDI-X consists of 50 items, of which 25 are related to PTG and the other 25 to PTD. Although the PTGDI-X includes five distinct domains [71] for PTG and PTD separately (relating to others: sense of concern and affinity with others; new possibilities: a new way discovered thanks to the difficult experience; personal strength: sense of self-reliance; spiritual and existential change: comprehension of spiritual matters; appreciation of life: awareness of life’s value), the overall scores for PTG and PTD can be used. In this study, we used only the overall PTG score. The respondents denoted their experience of perceived changes in the self on a scale ranging from 0 (“I did not experience this change”) to 5 (“I experienced this change to a great degree”). The results were calculated separately for the PTG and PTD scales, and separately for each subscale. Higher scores indicated a greater growth or depreciation. In the present study, Cronbach’s α coefficient for the PTG scale was excellent (α = 0.97), similar to the outcomes obtained in other Polish studies [72].

### 2.3. World Assumption Scale (WAS)

The World Assumption Scale developed by Janoff-Bulman [34], in its Polish adaptation by Załuski and Gajdosz [73], consists of 32 statements used to assess the beliefs about the world after experiencing a critical life event. The scale contains eight assumptions (benevolence of the impersonal world, benevolence of people, justice, controllability, randomness, self-worth, self-controllability, and luck). These assumptions are classified into three categories (benevolence of the world, meaningfulness, and worthiness of the self). The respondents gave their answers on a 6-point Likert scale from 1 (“I strongly disagree”) to 6 (“I strongly agree”). Some items required reverse scoring. The scores can be calculated for each of the 8 subscales (scores are in the range of 8–24 points), or as the total sum for the three categories. The higher the score, the stronger the belief. Cronbach’s α for the subscales usually range from 0.73 to 0.84 [74,75]. In the present study, the reliability of the three categories was as follows: benevolence of the world (α = 0.87), meaningfulness (α = 0.70), and worthiness of the self (α = 0.84).

### 2.4. Event-Related Rumination Inventory (ERRI)

The Event-related Rumination Inventory, developed by Cann et al. [54], in its Polish adaptation by Ogińska-Bulik and Juczyński [76], is a measure that assesses two styles of rumination: intrusive and deliberate. The first style concerns obtrusive and unintentional ruminations connected to distress continuing over a period of time (e.g., “Thinking about this event made me relive it”). The second style relates to reflective and intentional ruminations associated with comprehension of the difficult situation and problem solving (e.g., “I was wondering what significance an experienced event has for my future”). The inventory includes 20 items. Each scale contains 10 items. Respondents were asked to answer how often (if at all) they experienced the situations described in the inventory within a few weeks after a particularly difficult event. They assessed the statements on a 4-point Likert scale from 0 (“not at all”) to 3 (“often”). The results were calculated for both scales separately. The obtained internal compliance indices, which were measured using Cronbach’s α coefficients, were usually high and amounted to: 0.94–0.96 for the intrusive rumination scale and 0.88–0.92 for the reflective rumination scale [22,76]. In the present study, the reliability for both scales was excellent: α = 0.95 (intrusive rumination) and α = 0.90 (reflective rumination).

### 2.5. Statistical Analysis

All data were investigated with IBM SPSS Statistics version 20 (Armonk, NY, USA). The variables of world assumptions, post-traumatic growth, and rumination were checked for the normality of distribution. The values of skewness and kurtosis between ±2 were accepted as indicators of a relatively normal distribution [77]. The variance inflation factor (VIF) was introduced to measure the degree of collinearity between independent variables in a multiple linear regression model. A conservative cut-off for the presence of multicollinearity was adopted as a VIF value of over 2.5 [78]. Likewise, we calculated a tolerance value and assumed the generally recommended value between 0.1 to 0.2 as an indicator of a collinearity problem [79]. The presence of potential multivariate outliers was checked with the use of the Mahalanobis distance and Cook’s distance. The Mahalanobis distance was calculated using the chi-square (χ^2^) test with 10 degrees of freedom and a rigorous probability estimate of *p* < 0.001 [80]. A Cook’s distance measure close to 1 was regarded as a sign of influential observations [81].

Stepwise regression was used to assess the association between independent (dimensions of world assumption and rumination) and dependent (post-traumatic growth) variables after adjusting for the most common confounders, including sex, age, marital status, education level, and timing of cancer diagnosis (months). Potential confounders were included in the first step, and the dimensions of world assumption and rumination were placed in the second step. Differences between the sexes are well documented in cancer epidemiology [82], and age was found to act as a confounding factor in different studies [83]. Both marital status [84] and educational level [85] are considered important factors for the stage of cancer at diagnosis. Finally, the timing of the cancer diagnosis is not indifferent among cancer survivors in the context of their quality of life [86].

The statistical analyses were computed using the PROCESS macro 3.4. with Model 1. The indirect effect at various levels of the moderator was tested with bootstrap confidence intervals at 95% (5000 samples) [87].

## 3. Results

### 3.1. Descriptive Statistics

The descriptive statistics (mean, standard deviation, skewness, and kurtosis) for world assumptions, post-traumatic growth, and rumination are listed in Table 2.

The skewness and kurtosis values ranged from −1.171 to −0.033, indicating a relatively normal distribution within the acceptable ±2. Consequently, a Pearson correlation analysis was performed.

### 3.2. Correlations

The results of the Pearson analysis (Table 3) showed statistically significant (*p* < 0.001) correlations between post-traumatic growth, three dimensions of world assumptions, and intrusive and deliberate rumination. In line with past research, post-traumatic growth correlated positively with benevolence of the world (H1), meaningfulness (H1), and worthiness of the self (H1). It was also negatively associated with intrusive rumination (H2) and positively with deliberate rumination (H2). The Pearson correlation coefficients obtained in the study were small and large.

### 3.3. Multicollinearity and Confounding Variables

A VIF of 1.10–2.22 was obtained from the multiple regression analysis. Hence, it can be concluded that the predictors were averagely correlated with one another and there was a lack of collinearity. The values for tolerance were higher than 0.2 and ranged from 0.450 to 0.907, confirming that multicollinearity was not a problem in this study. The Mahalanobis distance method showed the presence of two outliers in the data (*p* = 0.000003 and *p* = 0.000004). As the results with and without the outliers were very similar, suggesting that the outliers were not influential and had little effect, we decided not to remove them. The Cook’s distance values ranged between 0.000 and 0.055, corroborating that there were no unusual observations in the sample. The regression analysis for confounding effects confirmed that four of five of the variables included in Step 1 did not act as real confounders: sex (*β* = 0.039, *t* = 0.652, *p* = 0.515), age (*β* = 0.043, *t* = 0.725, *p* = 0.470), marital status (*β* = 0.096, *t* = 1.573, *p* = 0.117), and education level (*β* = 0.028, *t* = 0.463, *p* = 0.644). However, time from diagnosis was a significant confounder (*β* = −0.161, *t* = −2.642, *p* = 0.009). Both the not relevant confounders and time from diagnosis explained only 4.0% of the variance (*R^2^* = 0.040). The dimensions of world assumptions, and the intrusive and deliberate rumination from Step 2, explained an additional 34.90% of the remaining variance.

### 3.4. Moderation

The findings of the six simple separate moderation models (Model 1), gained using bias-corrected bootstrap confidence intervals (95%) and 5000 bootstrap samples, showed a good fit to the data in the following models: (1) benevolence of the world, intrusive rumination, post-traumatic growth; (2) worthiness of the self, intrusive rumination, post-traumatic growth; and (3) meaningfulness, deliberate rumination, post-traumatic growth. In the other models, the ruminations did not have a moderator effect.

**Figure 1:**
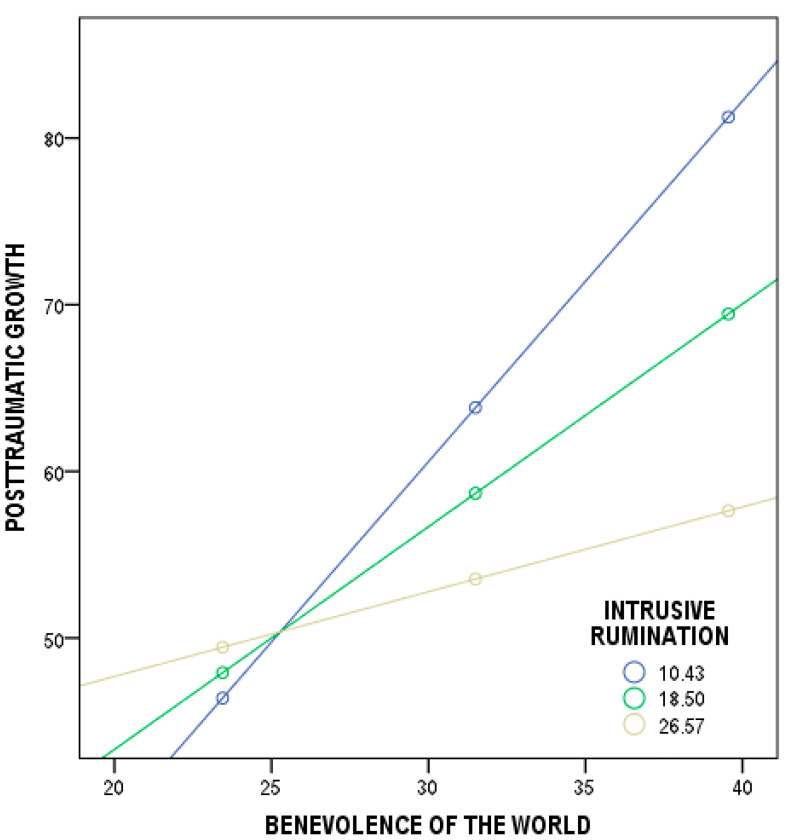
Plot of the slope analysis for the moderating effect of intrusive rumination in the relationship between benevolence of the world and post-traumatic growth.

With respect to benevolence of the world and post-traumatic growth, the overall model was found to be significant (*F*(3, 211) = 15.06, *p* < 0.001). Three predictors (benevolence of the world, intrusive rumination, and their interaction) predicted cancer patients’ scores in post-traumatic growth and explained 17% (*R^2^* = 0.17) of the variance. More specifically, the regressions were statistically significant for: (1) benevolence of the world and post-traumatic growth (*b* = 3.23, *t*(211) = 5.38, *p* < 0.001, 95% CI [2.0509;4.4173]); (2) intrusive rumination and post-traumatic growth (*b* = 2.59, *t*(211) = 2.72, *p* < 0.001, 95% CI [0.7191;4.4691]); and (3) the total interaction coefficient of the model tested (*b* = −0.10, *t*(211) = −3.42, *p* < 0.001, 95% CI [−0.1616;−0.0436]). The outcomes show that the moderation effect was significant for two conditions of intrusive rumination: low—10.4268 (*b* = 2.16, *t*(211) = 6.22, *p* < 0.001, 95% CI [1.4786;2.8503]); and medium—18.4977 (*b* = 1.33, *t*(211) = 5.06, *p* < 0.001, 95% CI [0.8168;1.8561]). The moderation effect was not significant for the high condition of intrusive rumination—26.5685 (*b* = 0.50, *t*(211) = 1.38, *p* = 0.167, 95% CI [−0.2146;1.2315]) (Figure 1). The three levels of both moderators were computed using the common recommendation of the mean as the medium level, plus one standard deviation as the high level, and minus one standard deviation as the low level [88].

**Figure 2:**
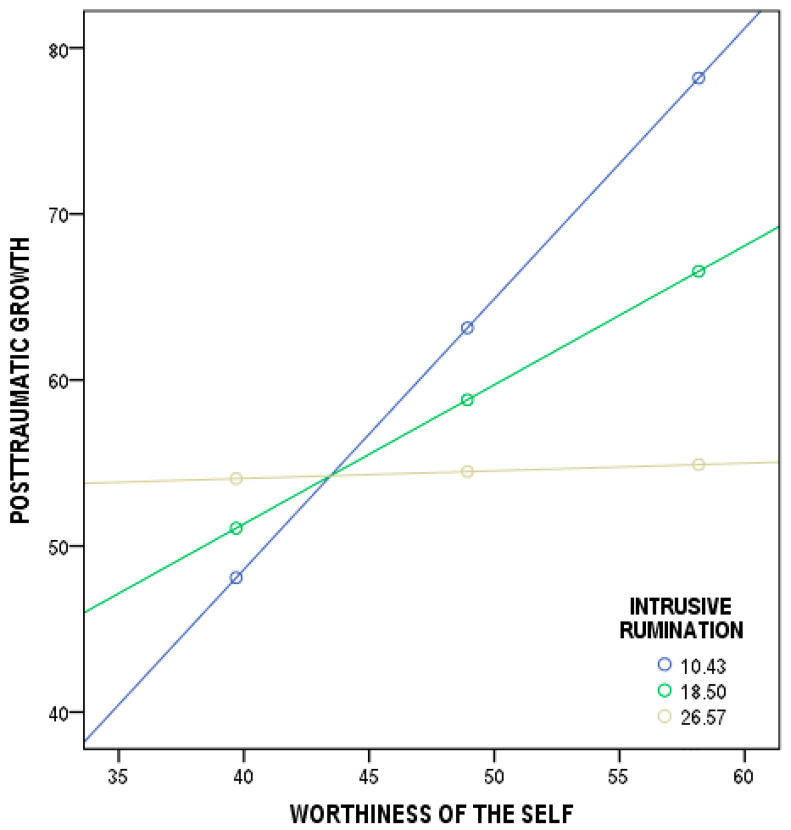
Plot of the slope analysis for the moderating effect of intrusive rumination in the relationship between worthiness of the self and post-traumatic growth.

With regard to worthiness of the self and post-traumatic growth, the overall model was significant (*F*(3, 211) = 12.97, *p* < 0.001). Three variables (worthiness of the self, intrusive rumination, and their interaction) predicted cancer patients’ scores in post-traumatic growth and explained 15% (*R^2^* = 0.15) of the variance. More explicitly, the regressions were statistically significant for: (1) worthiness of the self and post-traumatic growth (*b* = 2.65, *t*(211) = 5.37, *p* < 0.001, 95% CI [1.6784;3.6253]); (2) intrusive rumination and post-traumatic growth (*b* = 4.26, *t*(211) = 3.23, *p* < 0.001, 95% CI [1.6629;6.8622]); and (3) the total interaction coefficient of the model tested (*b* = −0.09, *t*(211) = −3.75, *p* < 0.001, 95% CI [−0.1496;−0.0465]). The findings show that the moderation effect was significant for two conditions of intrusive rumination: low—10.4268 (*b* = 1.62, *t*(211) = 5.74, *p* < 0.001, 95% CI [1.0698;2.1885]); and medium—18.4977 (*b* = 0.83, *t*(211) = 3.53, *p* < 0.001, 95% CI [0.3702;1.3048]). The moderation effect was not significant for the high condition of intrusive rumination—26.5685 (*b* = 0.04, *t*(211) = 0.13, *p* = 0.895, 95% CI [−0.6399;0.7316]) (Figure 2).

**Figure 3:**
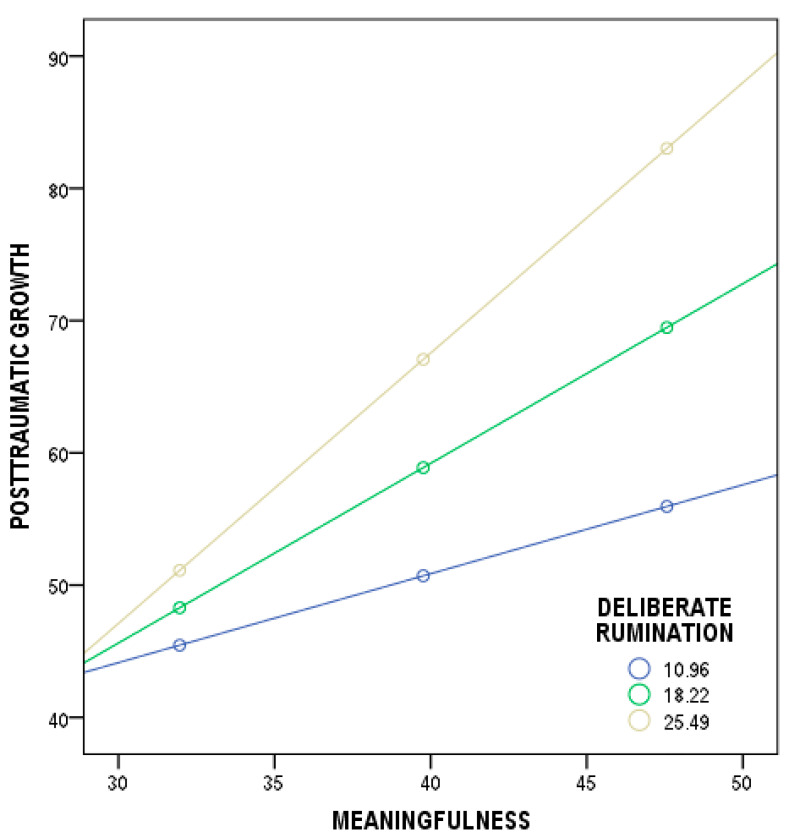
Plot of the slope analysis for the moderating effect of deliberate rumination in the relationship between meaningfulness and post-traumatic growth.

Referring to meaningfulness and post-traumatic growth, the overall model was significant (*F*(3, 211) = 16.52, *p* < 0.001). Three variables (meaningfulness, deliberate rumination, and their interaction) predicted cancer patients’ scores in post-traumatic growth and explained 19% (*R^2^* = 0.19) of the variance. Although the regressions of meaningfulness and post-traumatic growth (*b* = −0.36, *t*(211) = −0.46, *p* = 0.642, 95% CI [−1.9012;1.1759]), as well as deliberate rumination and post-traumatic growth (*b* = −2.62, *t*(211) = −1.70, *p* = 0.089, 95% CI [−5.6648;0.4073]), were not statistically significant, the total interaction coefficient of the model tested (*b* = 0.09, *t*(211) = 2.44, *p* = 0.015, 95% CI [0.0183;0.1706]) was significant. The results confirm that the moderation effect was significant for two conditions of deliberate rumination: medium—18.2233 (*b* = 1.35, *t*(211) = 5.01, *p* < 0.001, 95% CI [0.8247;1.8918]); and high—25.4884 (*b* = 2.04, *t*(211) = 5.54, *p* < 0.001, 95% CI [1.3173;2.7713]). The moderation effect was not significant for the low condition of deliberate rumination—10.9582 (*b* = 0.67, *t*(211) = 1.63, *p* = 0.102, 95% CI [−0.1360;1.4803]) (Figure 3).

## 4. Discussion

Considering the findings of the current study, it can be assumed that belief in the benevolence of the world or people, and a sense of meaning among patients diagnosed with cancer, coexists with post-traumatic growth. At the same time, patients who have the subjective experience of positive change as an outcome of traumatic strain declare lower levels of unintentional ruminations connected to distress, and higher levels of reflective ruminations associated with comprehension of the difficult situation.

Positive, albeit weak, correlations between the dimensions of world assumptions and post-traumatic growth (H1) are consistent with those previously obtained in various populations. For example, Lilly and Pierce [89] found that people who believe that the world is benevolent, things occur for a reason, and the self is worthy of respect display lower anxiety or depressive symptoms than their counterparts who present assumptions of the world as a dangerous place and a scarce sense of self-worth. This is possibly due to the cognitive engagement in the processing of a traumatic experience. It allows people to make the right changes in their lives, strengthen their personal self, and discover new possibilities [24]. In fact, theories of cognitive adaptation to trauma advance that retrospection and confrontation with crisis may lead to positive outcomes and greater post-traumatic growth. Salsman et al. [90] and Bayer et al. [91] noticed that people with higher levels of basic assumptions declared higher levels of post-traumatic growth. As Calhoun et al. [31] observed, change during or after a crisis is possible thanks to the cognitive and emotional work that consists of reconstructing basic assumptions about the world and the self.

The second hypothesis (H2) was also confirmed as post-traumatic growth correlated negatively with intrusive rumination and positively with deliberate rumination. This result may be due to the fact that both types of rumination have different functions in post-traumatic reactions [92]. Although there are some studies that show a lack of correlation [62,93,94], or even a positive correlation [62,92], between each of the two types of rumination and post-traumatic growth, there is also some empirical evidence that engaging in intrusive thoughts after a difficult situation is negatively associated with post-traumatic growth [71,93]. In fact, it was found that the occurrence of intrusive thoughts indicates difficulty in handling stressful events in a constructive way [71]. In other studies [93], ruminative brooding, which is conceptually similar to intrusive rumination [95,96], correlated negatively with post-traumatic growth. With respect to deliberate thinking, its positive association with post-traumatic growth is consistent with several studies where deliberate rumination had a positive correlation to or was a strong predictive factor of post-traumatic growth [62,77,92,97,98] and with Calhoun and Tedeschi’s theoretical model [99], which shows that positive change is an effect of purposeful rumination following the strive to comprehend a traumatic event.

Finally, the outcomes show that the level of post-traumatic growth resulting from world assumptions is significantly different at various levels of intrusive and deliberate rumination (H3). Based on these findings, it can be supposed that the relationship between benevolence of the world and post-traumatic growth is stronger when cancer patients express lower and medium levels of intrusive rumination than when cancer patients indicate higher levels of intrusive thoughts. Moreover, the relationship between worthiness of the self and post-traumatic growth is weakened by the intensity of intrusive thoughts. The higher the intrusive rumination declared by the cancer patients, the weaker the relationship between worthiness of the self and post-traumatic growth. Finally, the relationship between meaningfulness and post-traumatic growth is strengthened by the intensity of deliberate thoughts. The higher the deliberate rumination disclosed by the cancer patients, the stronger the relationship between meaningfulness and post-traumatic growth.

Considering that traumatic events often challenge one’s core beliefs about the world and themselves, individuals need to rethink their point of view before and after a difficult situation [94]. Negative and undesired thinking patterns after a crisis may lead to post-traumatic stress [100] because intrusive rumination makes people concentrate on the negative outcomes [101], especially soon after experiencing a traumatic event [102]. Over time, unwanted coping thoughts and challenges can lead people struggling with a difficult event to consciously reflect on what happened [103]. Thus, deliberate rumination and purposeful thinking seem to positively influence post-traumatic growth [20] because such growth involves considerable cognitive effort [103] and an active role in the process [53]. Other studies also showed that rumination interacted with some cognitive factors to predict negative expectancies [104] and depression symptoms [105,106]. When people have lower and medium levels of intrusive rumination, and higher levels of deliberate rumination, they present a lower intensity of pessimistic thoughts and lower negative memories [105]. This is consistent with some cognitive models and theories which suggest that self-focused attention may play a main role in impacting both adaptive and maladaptive responses [107].

## 5. Limitations

Despite its strengths, this study has also its limitations. The first limitation of the present research concerns the unequal proportion of men and women suffering from different categories of cancer, which makes a comparative gender analysis difficult. To achieve more objective conclusions, the research group should be more even in terms of sex. The second limitation relates to the variety of cancer types. As the sample consisted of patients with various forms of cancer, further studies could provide more homogenous groups with the analysis of potential confounding variables related to specific risk factors.

## 6. Conclusions

With respect to the results obtained in the study, although lower/medium intrusive rumination seemed to strengthen the relationship between benevolence of the world/worthiness of the self and post-traumatic growth, and higher deliberate rumination tends to reinforce the relationship between meaningfulness and post-traumatic growth, caution should be exercised in interpreting the findings. The relationship between world assumptions and post-traumatic growth is complex and depends on several factors, not only of a psychological nature, but also of a social and economic nature. Therefore, taking these variables into account could provide a broader perspective on the character of this association.

## Figures and Tables

**Table 1:** Demographics, marital status, education, and type of cancer (*N* = 215).

Variables	
**Age** (years)	M 37.74 (*SD* = 13.97)
**Sex**	
Female	92.60%
Male	7.40%
**Marital status**	
Married	30.20%
Informal relationship	31.20%
Single	33.50%
Divorced	5.10%
**Education**	
Secondary	38.60%
High	61.40%
**Type of Cancer**	
Lung	7.90%
Breast	42.30%
Pancreatic	5.60%
Esophageal	5.10%
Stomach	4.20%
Prostate	1.40%
Testicular	3.70%
Liver	7.40%
Gynecologic	22.30%

**Table 2:** Descriptive statistics of world assumptions, post-traumatic growth, and rumination (N = 215).

Variables	M	SD	Skewness	Kurtosis
Benevolence of the world	31.49	8.05	−0.151	−0.092
Meaningfulness	39.76	7.8	−0.211	−0.057
Worthiness of the self	48.92	9.23	−0.095	−0.344
Post-traumatic growth	59.11	33.86	−0.033	−1.171
Intrusive rumination	18.49	8.07	−0.077	−0.932
Deliberate rumination	18.22	7.26	−0.337	−0.843

**Table 3:** Pearson correlation coefficients between world assumptions, post-traumatic growth, and rumination (*N* = 215).

Variables	BW	ME	WS	PG	IR	DR
Benevolence of the world (BW)	1					
Meaningfulness (ME)	0.28 ***	1				
Worthiness of the self (WS)	0.70 ***	0.28 ***	1			
Post-traumatic growth (PG)	0.34 ***	0.34 ***	0.28 ***	1		
Intrusive rumination (IR)	−0.06	−0.04	−0.04	−0.15 *	1	
Deliberate rumination (DR)	0.01	0.04	0.03	0.24 ***	0.54 ***	1

* *p* < 0.05; *** *p* < 0.001.

## Data Availability

The datasets used during this study are available from the corresponding author.
